# The role of clinical phenotypes in decisions to limit life-sustaining treatment for very old patients in the ICU

**DOI:** 10.1186/s13613-023-01136-7

**Published:** 2023-05-10

**Authors:** Oded Mousai, Lola Tafoureau, Tamar Yovell, Hans Flaatten, Bertrand Guidet, Michael Beil, Dylan de Lange, Susannah Leaver, Wojciech Szczeklik, Jesper Fjolner, Akiva Nachshon, Peter Vernon van Heerden, Leo Joskowicz, Christian Jung, Gal Hyams, Sigal Sviri

**Affiliations:** 1 School of Computer Science and Engineering, The Hebrew University of Jerusalem, Givat Ram, Jerusalem, Israel; 2 Department of Anaesthesia and Intensive Care, Haukeland University Hospital, Bergen, Norway; 3 Sorbonne Université, INSERM, Institut Pierre Louis d′Epidémiologie et de Santé Publique, AP-HP, Hôpital Saint Antoine, service MIR, Paris, France; 4 Department of Medical Intensive Care, Faculty of Medicine, Hebrew University and Hadassah University Medical Center, Jerusalem, Israel; 5 Department of Intensive Care Medicine, University Medical Center, University Utrecht, Utrecht, The Netherlands; 6 General Intensive Care, St George’s University Hospitals NHS Foundation Trust, London, UK; 7 Center for Intensive Care and Perioperative Medicine, Jagiellonian University Medical College, Kraków, Poland; 8 Department of Anaesthesia and Intensive Care, Viborg Regional Hospital, Viborg, Denmark; 9 General Intensive Care Unit, Department of Anaesthesiology, Critical Care and Pain Medicine, Faculty of Medicine, Hebrew University and Hadassah University Medical Center, Jerusalem, Israel; 10 Division of Cardiology, Department of Cardiology, Pulmonology and Vascular Medicine, Faculty of Medicine, Heinrich-Heine-University, Moorenstraße 5, 40225 Düsseldorf, Germany

**Keywords:** Geriatric patients, Intensive care, Phenotypes, Withdrawing, Withholding

## Abstract

**Background:**

Limiting life-sustaining treatment (LST) in the intensive care unit (ICU) by withholding or withdrawing interventional therapies is considered appropriate if there is no expectation of beneficial outcome. Prognostication for very old patients is challenging due to the substantial biological and functional heterogeneity in that group. We have previously identified seven phenotypes in that cohort with distinct patterns of acute and geriatric characteristics. This study investigates the relationship between these phenotypes and decisions to limit LST in the ICU.

**Methods:**

This study is a post hoc analysis of the prospective observational VIP2 study in patients aged 80 years or older admitted to ICUs in 22 countries. The VIP2 study documented demographic, acute and geriatric characteristics as well as organ support and decisions to limit LST in the ICU. Phenotypes were identified by clustering analysis of admission characteristics. Patients who were assigned to one of seven phenotypes (*n* = 1268) were analysed with regard to limitations of LST.

**Results:**

The incidence of decisions to withhold or withdraw LST was 26.5% and 8.1%, respectively. The two phenotypes describing patients with prominent geriatric features and a phenotype representing the oldest old patients with low severity of the critical condition had the largest odds for withholding decisions. The discriminatory performance of logistic regression models in predicting limitations of LST after admission to the ICU was the best after combining phenotype, ventilatory support and country as independent variables.

**Conclusions:**

Clinical phenotypes on ICU admission predict limitations of LST in the context of cultural norms (country). These findings can guide further research into biases and preferences involved in the decision-making about LST.

*Trial registration* Clinical Trials NCT03370692 registered on 12 December 2017.

## Background

Decisions to withhold or withdraw life-sustaining treatment (LST) in the intensive care unit (ICU) are considered appropriate if there is no reasonable expectation of beneficial outcome [1]. However, the evaluation of prognostic information and benefit of critical care for the individual patient varies depending on a number of factors which can be related or unrelated to the individual patient, such as cultural norms and resource constraints [2–6]. Patient-related factors comprise the severity of the acute illness, comorbidities and, notably, old age [7, 8]. However, predicting outcome and its benefit for very old patients and making appropriate decisions about LST constitute a major challenge due to the heterogeneity of multimorbidity and the variable perception of functional impairments at an advanced age [9–11]. This has resulted in a substantial variability of decisions to withhold or withdraw LST in critical care [3, 12, 13].

We have recently identified distinct phenotypes of very old patients (age ≥ 80 years) from the multinational VIP2 study cohort by using clustering analysis of clinical characteristics available on admission to the ICU [14, 15]. This method provided the opportunity to explore complex patterns of clinical features to draw a nuanced picture of this patient population with regard to prognosis [16]. In a subgroup of VIP2 patients without limitations of LST, short-term mortality was found to be highest (up to 57% within 30 days) for phenotypes with marked geriatric features, i.e., frailty, multimorbidity and functional or cognitive impairments. In contrast, 30-day mortality in a phenotype composed of nonagenarians with low sequential organ failure assessment (SOFA) scores was less than 10%, which defied traditional views on the benefit of LST in that age group [16].

This new study sets out to investigate whether the decisions to withhold or withdraw LST in the ICU depend on the patients' clinical phenotype in the VIP2 cohort. We compare the influence of phenotypes on these decisions with the impact of the cultural context (country) which was shown to play a significant role in a similar cohort [12]. This analysis of practice patterns is needed to support timely discussions with patients and their families about care trajectories for critical and potentially terminal conditions [17].

## Methods

The Very elderly Intensive care Patient (VIP)—2 study was a prospective observational study to examine the influence of geriatric characteristics on survival in patients aged 80 years or older admitted with acute conditions to ICUs in 22 countries [14]. The participating ICUs recruited consecutive patients who met the above demographic and clinical criteria during any 6-month period between May 2018 and May 2019. National coordinators obtained ethics committee approval in their respective countries. Case report forms and the database were hosted on a secure server located on the campus of Aarhus University (Denmark).

Clustering analysis was applied to the VIP2 study cohort to delineate groups (phenotypes) of patients with similar demographic (age, gender, residence), acute (SOFA score and subscores) and geriatric characteristics (frailty, multimorbidity and polypharmacy, functional and cognitive impairments) recorded on admission to the ICU [15]. Decisions to limit LST were recorded as withholding or withdrawing LST in the VIP2 study. Sensitivity analyses were performed with respect to the inclusion of patients with limitations of LST and the number of phenotypical categories [15].

**Fig. 1:**
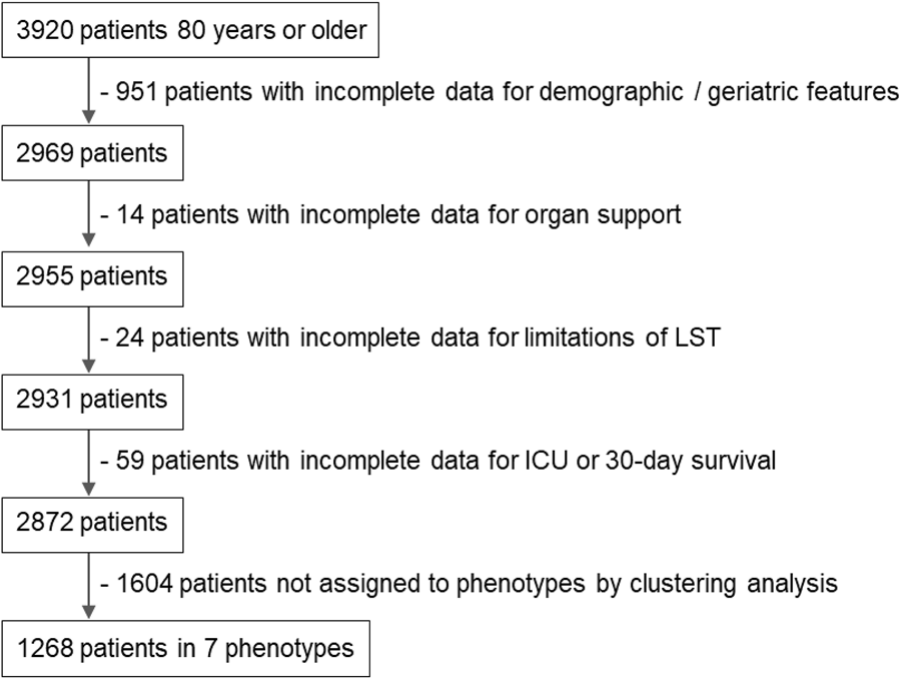
Flowchart for the study sample obtained from the VIP2 study cohort

This new descriptive study includes all patients from the VIP2 cohort who were classified into one of seven distinct phenotypes and who stayed in ICU for more than 1 h. The flowchart for obtaining this sample is depicted in Fig. 1.

Descriptive characteristics are reported as median with inter-quartile range (IQR) for continuous variables and proportions (percentages) for nominal variables. Odds ratios with 95% confidence intervals were calculated for binary outcome variables, i.e. either withholding or withdrawing LST, for each phenotype with the phenotype having the highest rate for these outcomes as reference. One-way ANOVA test was used to examine differences of continuous variables and Fisher’s exact test for nominal variables. The area under the receiver-operating characteristic (AUROC) curve was determined for logistic regression models to assess their discriminatory performance for the binary classification of outcome. Statistical analyses were performed using R (version 4.1.1, www.r-project.org) and Python 3 (Python Software Foundation, Beaverton, OR, USA).

## Results

This study included 1268 patients from the VIP2 study cohort with or without limitations of LST who were assigned to one of seven phenotypes [15]. The incidence of decisions to withhold or withdraw LST was 26.5% and 8.1%, respectively. Overall mortality in this population was 17.7% in ICU and 27.1% within 30 days. The mortality at 30 days after withholding or withdrawing LST was 34.5% and 88.3%, respectively.

The demographic and clinical characteristics of phenotypes are shown in Table 1. Phenotypes A and G represent the extreme ends of the spectrum of the SOFA score, most geriatric features and mortality. Mortality in the ICU was significantly higher for phenotypes A, B and C after decisions to withhold LST. Statistically significant differences in 30-day mortality were detected for phenotypes A–E but not for the geriatric phenotypes F and G (Table 1).

**Table 1:** Demographic and clinical characteristics of phenotypes

Variable	Phenotype							*p*-value
	A-very low SOFA	B-respirat. failure	C-oldest old	D-moderate SOFA	E-renal failure	F-geriatric, low SOFA	G-geriatric, high SOFA	
Number of patients	244	247	134	252	143	106	142	
Age-median (IQR)	83 (81–85)	83 (81–85)	92 (90–94)	83 (81–85)	83 (81–85)	84 (82–86)	85 (83–89)	< 0.001
Gender-number (% female)	123 (50%)	116 (47%)	83 (61%)	91 (36%)	45 (31%)	68 (64%)	74 (52%)	< 0.001
Admission reason-number (%)								
Respiratory/cardiovascular	58 (23%)	187 (75%)	80 (59%)	119 (47%)	61 (42%)	63 (59%)	73 (51%)	< 0.001
Sepsis	8 (3%)	16 (6%)	5 (3%)	39 (15%)	26 (18%)	9 (8%)	42 (29%)	< 0.001
Emergency surgery	51 (20%)	20 (8%)	21 (15%)	56 (22%)	11 (7%)	11 (10%)	14 (9%)	< 0.001
SOFA score-median (IQR)	1 (0–2)	4 (3–4)	4 (2–5)	7 (7–9)	6 (5–8)	3 (2–4)	11 (10–12)	< 0.001
Geriatric features^a^ median (IQR)								
CFS	3 (2–3)	4 (3–4)	4 (3–5)	3 (2–4)	4 (3–4)	6 (6–7)	7 (6–7)	< 0.001
Katz score	6 (6–6)	6 (6–6)	5 (4–6)	6 (6–6)	6 (5–6)	3 (2–4)	1 (0–2)	< 0.001
IQCODE	3.1 (3–3.2)	3.1 (3–3.3)	3.4 (3.1–3.9)	3.1 (3–3.3)	3.2 (3–3.4)	3.7 (3.2–4)	4.7 (4.1–5)	< 0.001
CPS	7 (5–10)	12 (9–14)	9 (6–12)	9 (6–12)	12 (9–16)	16 (14–20)	11 (8–14)	< 0.001
Interventions-number (%)								
Invasive ventilation	38 (15%)	88 (35%)	32 (23%)	191 (75%)	33 (23%)	24 (22%)	121 (85%)	< 0.001
Vasopressors	50 (20%)	66 (26%)	54 (40%)	249 (98%)	74 (51%)	39 (36%)	136 (95%)	< 0.001
Renal replacement therapy	4 (1%)	4 (1%)	0	27 (10%)	62 (43%)	5 (4%)	26 (18%)	< 0.001
Limitations of LST-number (%)								
LST withheld	26 (10%)	59 (23%)	53 (39%)	68 (27%)	32 (22%)	40 (37%)	58 (40%)	< 0.001
LST withdrawn	5 (2%)	25 (10%)	7 (5%)	31 (12%)	10 (7%)	9 (8%)	16 (11%)	< 0.001
LST withheld and withdrawn	3 (1%)	15 (6%)	5 (3%)	22 (8%)	9 (6%)	6 (5%)	12 (8%)	0.003
Length of stay in ICU (days)-median (IQR)	2.4 (1.1–4)	5 (2.2–8.5)	3 (1.1–5)	6 (3–12)	5 (2.7–7.9)	3 (1.9–6)	6 (2.5–13)	< 0.001
Died in ICU-number (%)								
All	12 (5%)	30 (12%)	18 (13%)	51 (20%)	22 (15%)	10 (9%)	81 (57%)	< 0.001
After LST withheld	6 (23%)	17 (29%)	13 (25%)	27 (40%)	13 (41%)	5 (13%)	35 (60%)	< 0.001
(*p* value^b^)	(0.004)	(0.017)	(0.031)	(0.29)	(0.067)	(1.0)	(0.58)	
Died within 30 days-number (%)								
All	21 (9%)	52 (21%)	42 (31%)	74 (29%)	33 (23%)	25 (24%)	96 (68%)	< 0.001
After LST withheld	11 (42%)	25 (42%)	27 (51%)	39 (57%)	19 (59%)	13 (32%)	44 (76%)	< 0.001
(*p* value^b^)	(< 0.001)	(0.004)	(< 0.001)	(0.003)	(< 0.001)	(0.13)	(0.13)	

^a^CFS—clinical frailty scale, Katz score to assess functional ability by activities of daily living, IQCODE—informant questionnaire on cognitive decline in the elderly, CPS—comorbidity and polypharmacy score

^B^for comparison with patients without limitations of LST

Table 2 shows the distribution of phenotypes and the incidence of limitations of LST in the patient cohorts from countries which contributed more than 3% of the study population each.

**Table 2:** Characteristics of patient cohorts from countries which contributed more than 3% of the study population

Country	Number of patients	Withhold LST (%)	Withdraw LST (%)	Number of patients in specific phenotypes (%)						
				A	B	C	D	E	F	G
1	217	69 (32)	26 (12)	26 (12)	60 (28)	28 (13)	32 (15)	37 (17)	18 (8)	16 (7)
2	205	20 (10)	13 (6)	61 (30)	57 (28)	20 (10)	28 (14)	30 (15)	9 (4)	0 (0)
3	138	26 (19)	11 (8)	49 (36)	25 (18)	4 (3)	28 (20)	12 (9)	19 (14)	1 (1)
4	105	60 (57)	14 (13)	30 (29)	26 (25)	4 (4)	31 (30)	10 (10)	3 (3)	1 (1)
5	92	19 (21)	4 (4)	2 (2)	5 (5)	3 (3)	21 (23)	9 (10)	2 (2)	50 (54)
6	79	35 (44)	6 (8)	6 (8)	6 (8)	16 (20)	21 (27)	5 (6)	17 (22)	8 (10)
7	76	13 (17)	7 (9)	18 (24)	10 (13)	10 (13)	8 (11)	8 (11)	11 (14)	11 (14)
8	64	11 (17)	6 (9)	6 (9)	15 (23)	10 (16)	16 (25)	5 (8)	5 (8)	7 (11)
9	55	5 (9)	0 (0)	5 (9)	4 (7)	5 (9)	14 (25)	2 (4)	1 (2)	24 (44)
10	54	28 (52)	5 (9)	8 (15)	12 (22)	9 (17)	15 (28)	6 (11)	3 (6)	1 (2)

Phenotypes F and G and the group of oldest old patients (phenotype C) were found to be associated with the highest rates and largest odds for withholding decisions (Tables 1, 3). Phenotypes F and C did not differ significantly from phenotype G which had the highest overall rate for limitations of LST and served as reference. Regarding withdrawal of LST, phenotype A showed the smallest odds that differed significantly from the reference phenotype G (Table 3).

**Table 3:** Odds ratios (OR) with 95% confidence intervals for decisions to limit LST

Phenotype	Withhold LST		Withdraw LST	
	OR	*p* value	OR	*p* value
A	0.17 (0.10–0.29)	< 0.001	0.16 (0.053–0.43)	< 0.001
B	0.45 (0.29–0.71)	< 0.001	0.89 (0.46–1.8)	0.72
C	0.95 (0.58–1.5)	0.83	0.43 (0.16–1.1)	0.086
D	0.54 (0.35–0.83)	0.0049	1.1 (0.59–2.1)	0.76
E	0.42 (0.25–0.70)	< 0.001	0.59 (0.25–1.3)	0.21
F	0.88 (0.52–1.5)	0.62	0.73 (0.30–1.7)	0.47
G	1 (reference)		1 (reference)	

To investigate the relationship between phenotypes and limitations of LST in more detail, we examined the odds for withholding further LST in patients during noninvasive and invasive ventilation. Patients on noninvasive ventilation in phenotype D and patients on invasive ventilation in phenotype B had significantly lower odds than the reference phenotype G for withholding decisions when treated at these levels of organ support (Table 4). Of note, we did not perform a similar analysis for withdrawing decisions due to the small number of patients with that type of decision.

**Table 4:** Odds ratios (OR) with 95% confidence intervals for decisions to withhold LST in patients on noninvasive and invasive ventilation

Phenotype	Noninvasive ventilation		Invasive ventilation	
	OR	*p* value	OR	*p* value
A	0.22 (0.041–1.2)	0.086	0.46 (0.17–1.3)	0.18
B	0.37 (0.15–0.94)	0.054	0.35 (0.16–0.77)	0.011
C	1.1 (0.41–3.1)	1.0	1.01 (0.41–2.5)	1.0
D	0.30 (0.1–0.89)	0.039	0.59 (0.334–1.03)	0.078
E	0.33 (0.094–1.1)	0.12	0.42 (0.14–1.3)	0.16
F	0.71 (0.25–2.0)	0.59	2.2 (0.87–5.4)	0.13
G	1 (reference)		1 (reference)	

**Fig. 2:**
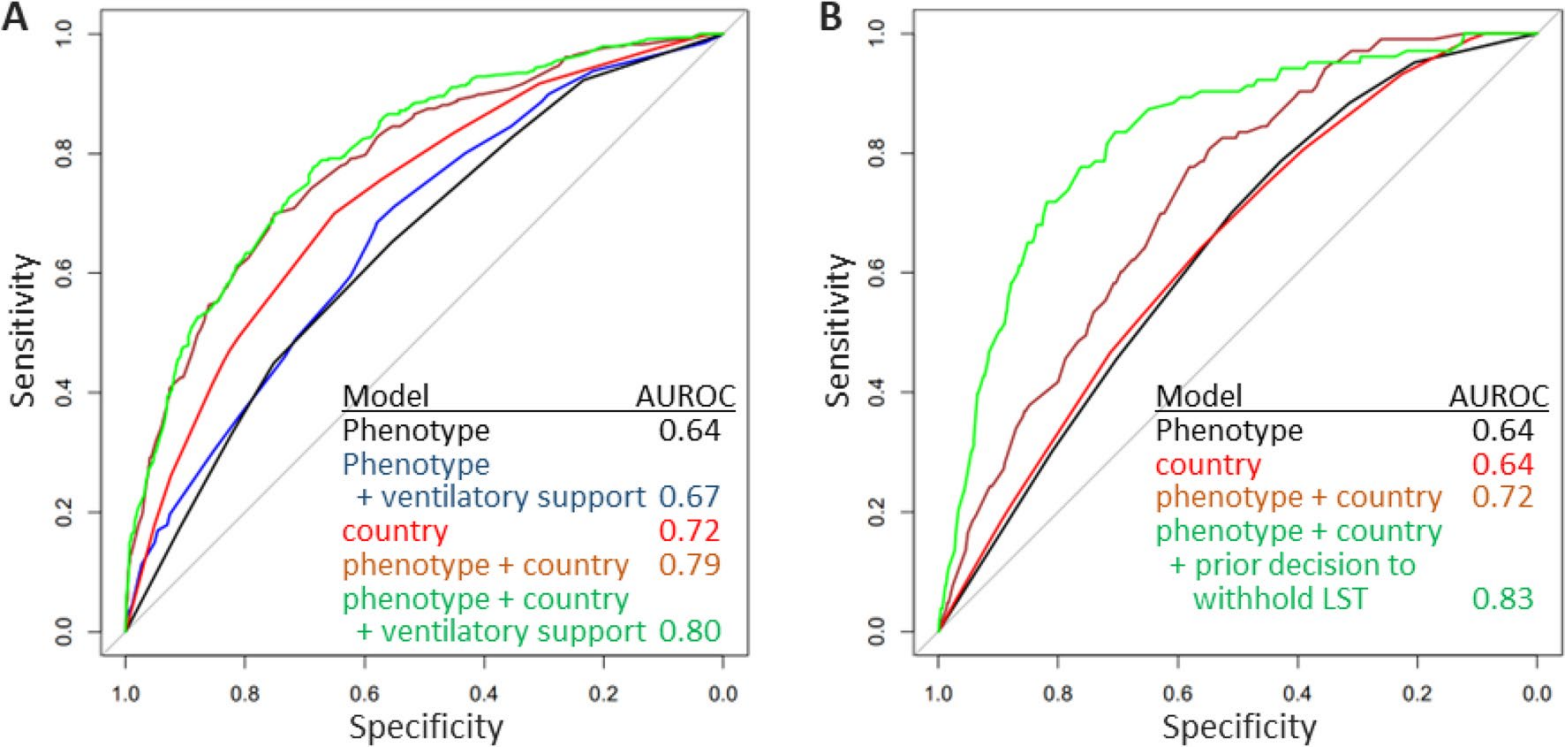
Discriminatory performance of logistic regression models, as depicted by receiver-operating characteristic (ROC) curves, for decisions to withhold (**A**) or withdraw (**B**) of LST

Next, we compared the discriminatory performance of logistic regression models based on phenotype, cultural contest (country), ventilatory support and the prior occurrence of withholding decisions to predict limitations of LST. Figure 2 shows the receiver operating characteristic curves and AUROC data for these models. Using phenotype or country alone did not yield good discrimination, i.e. AUROC values were below 0.8 for both types of decisions. A better discrimination was achieved by combining phenotype with country. Adding the history of withholding decisions resulted in a good discrimination with an AUROC value of 0.83 for decisions to withdraw LST (Fig. 2).

## Discussion

Limiting LST can be an important step to adjust the extent of critical care to the individual needs of patients. Due to the uncertainty about beneficial outcome, notably in very old patients, there is no evidence-based framework to guide these decisions. A more detailed understanding of the involved factors can increase the awareness to biases and may reduce the variability of decision-making [13]. In this context, the objective of this study was to investigate the role of clinical phenotypes for decisions to withhold or withdraw LST in the VIP2 study [14, 15]. These phenotypes represent combinations of demographic, acute and geriatric characteristics on admission to the ICU and are available for early discussions about likely trajectories in critical care.

Two of the phenotypes (F, G) are characterised by enhanced geriatric features. Phenotype C includes the oldest old patients, but without prominent geriatric characteristics and with only moderate SOFA scores. Importantly, the largest odds for decisions to withhold LST were found in these three phenotypes. This confirms previous studies showing an association of such decisions with the perception of poor performance status [7]. Although frailty and other geriatric impairments were shown to correlate with worse survival and functional outcome [18–22], there is no strong evidence for that with respect to age itself [23]. The new findings in this study suggest a propensity among medical professionals to limit the perceived burden of interventional therapies for the oldest old, independently of acute and chronic conditions. Importantly, mortality after 30 days was not significantly increased for the geriatric phenotypes F and G after withholding LST. This indicates coherence of predictions with the actual outcome in these phenotypes. However, there was a significant increase of mortality after withholding LST in phenotype C questioning the value of the above approach for this particular group of oldest old patients.

For patients on ventilatory support, the nongeriatric phenotypes B and D had a lower probability of withholding additional organ support. Patients in both phenotypes scored high for the respiratory component of the SOFA score on admission to ICU [15]. Thus, ventilatory support was one if not the main reason for admission to the ICU and continuation of organ support until remission of respiratory failure might have been a major objective. This reasoning, however, is not applicable for phenotype G which had the highest rate of invasive ventilation, but on a background of enhanced geriatric characteristics, which eventually led to a higher rate of decisions to limit LST.

We have recently examined the relationship between single patient characteristics (age, gender, SOFA score, single geriatric features) and decisions to limit LST for the VIP2 patient cohort [24]. There was no individual characteristic with meaningful discrimination for withholding decisions, i.e. AUROC values greater than 0.6. The small increment in discrimination gained by using phenotypes instead of single features illustrates both the complexity of choosing patients for withholding decisions and the need for additional information to predict these decisions with better accuracy. Regarding withdrawal of LST in that previous study [24], the SOFA score had the largest influence on these decisions with an AUROC value of 0.66. This level of discrimination is in the range of that of the phenotype-based model in the new study and reflects the prominent role of the SOFA score for delineating phenotypes with regard to withdrawing decisions [15]. This particularly applies to phenotype A with the lowest SOFA score and the lowest rate and odds for withdrawing LST.

What could be the additional information required for predicting limitations of LST more accurately? Candidate parameters are cultural norms and the response to treatment or the lack thereof as well as the occurrence of adverse events. Moreover, fluctuating resource constraints and preferences of individual stakeholders may have an additional impact on decision-making [25–27]. Although these parameters were not explicitly documented in the VIP2 study, we approximated cultural norms by the geographic location (country) of the participating ICUs and showed differences for the incidence of limitations of LST between countries. Ventilatory support and decisions to withhold further LST were used as surrogate markers for assessing the course of critical care in the ICU. In comparison to the patients' phenotype, country as a variable showed a better or at least similar discrimination for predicting withholding or withdrawing decisions. The combination of phenotype and country in a regression model led to a marked increase of discrimination. Adding the prior withholding of LST as an additional variable resulted in a good discrimination for predicting withdrawal of LST.

The above results emphasise the contribution of both patient-related factors and cultural norms to decisions about LST in very old patients. However, because discrimination was only moderate for our models, yet to be specified factors, such as variable characteristics of individual stakeholders, are likely to influence these decisions. Managing multiple factors influencing decision-making in critical care can be challenging. This has been illustrated by the controversies about triage during the COVID-19 pandemic, when the variable interpretation of patient-related information as well as diverse cultural attitudes led to variations of care [28, 29].

Our study has several limitations. The VIP2 study was not designed to analyse decisions to withhold or withdraw LST as outcome. Patients' preferences and other contextual data were not recorded and, thus, were not available for our analysis. Our study focused on phenotyped patients which constitute less than 50% of the eligible study population. The impact of variables other than phenotype on limitations of LST might be different in nonphenotyped patients. Moreover, follow-up was limited to survival at 30 days. Data on survival beyond that time and quality of life, which may be impaired by new disabilities and post-ICU syndrome [30], could further support the decision-making about LST in very old patients. Lastly, patients for the VIP2 study were mostly recruited in Europe [14]. Therefore, the findings on decisions to limit LST remain to be confirmed for other geographic regions [16].

## Conclusions

Our study demonstrates the role of clinical phenotypes for decisions to limit LST in very old ICU patients. Combining phenotypes with cultural factors and information about the course of critical care resulted in a good accuracy of predictive discrimination for withholding and withdrawing decisions. These findings can guide further research into biases and preferences involved in the decision-making about LST. Future studies should also analyse the impact of withholding LST on the self-perceived quality of life in ICU survivors to further personalise these decisions.

## Data Availability

The datasets analysed during the current study are not publicly available due to contractual restrictions but are available from the corresponding author on reasonable request.

## Abbreviations

AUROC: Area under the receiver-operating characteristic
ICU: Intensive care unit
LST: Life-sustaining treatment
SOFA: Sepsis-related organ failure assessment
